# ﻿A new synonym of *Enkianthusperulatus* (Ericaceae) in East Asia, based on morphological and molecular evidence

**DOI:** 10.3897/phytokeys.214.94294

**Published:** 2022-11-25

**Authors:** Hua Liang, Lu Jiang, Danqi Li, Yi Yang, Dengmei Fan, Zhiyong Zhang

**Affiliations:** 1 Laboratory of Subtropical Biodiversity, Jiangxi Agricultural University, Nanchang, Jiangxi, China; 2 Lushan Botanical Garden, Chinese Academy of Sciences, Jiujiang, Jiangxi, China; 3 Institute of Ecology, Jiangxi Agricultural University, Nanchang, Jiangxi, China

**Keywords:** *Enkianthuscalophyllus*, *Enkianthusperulatus*, morphology, phylogeny, synonym

## Abstract

*Enkianthuscalophyllus* was once treated as a synonym of *E.serrulatus*. However, field observations indicate that *E.calophyllus* is distinct from *E.serrulatus* but resembles *E.perulatus* in flowers, leaves, fruits and seeds. Hence, a taxonomic revision of these species was conducted based on morphological comparisons of flowers, leaves, fruits and seeds, as well as molecular analyses of nuclear ribosomal internal transcribed spacer (nrITS) and six plastid DNA markers (*psb*A-*trn*H, *rpl*32-*trn*L, *trn*L-*trn*F, *rps*16-*trn*Q, *psb*J-*pet*A and *mat*K). The morphological and molecular results reject the synonymization of *E.calophyllus* with *E.serrulatus*, and instead show it to be placed in a clade with *E.perulatus*. Based on molecular evidence and a reassessment of the morphology we synonymize *E.calophyllus* with the older name *E.perulatus*.

## ﻿Introduction

*Enkianthus* Lour. is a small genus in Ericaceae with about 12–17 species (Anderberg 1994; Kron et al. 2002; Fang and Stevens 2005). It is only distributed in East Asia, and most of its component species are in China and Japan. *Enkianthus* is ornamentally important for its elegant flowers and ecologically valuable because it always dominates in subtropical montane elfin forests (Hsu 1982). Accumulating molecular phylogenetic evidence suggests that *Enkianthus* is the first diverging lineage of Ericaceae (Kron and Chase 1993; Kron 1996; Morton et al. 1996; Anderberg et al. 2002; Kron et al. 2002), indicating that this genus is key to understand the evolution of Ericaceae.

Species in *Enkianthus* are shrubs or small trees, leaves blade serrate or subentire, inflorescences often umbels and racemes, corollas broadly campanulate to urceolate, capsule loculicidal, seeds often lamellate-winged (Fang and Stevens 2005). The species of *Enkianthus* vary in leaf texture, inflorescences structure, corolla shape, and anther morphology, pollen and seed (Cheng and Lai 1988; Anderberg 1994; Kron et al. 2002; Sarwar and Takahashi 2006). Infrageneric relationships of *Enkianthus* were studied by Anderberg (1994), who proposed a classification comprising four sections (sect. Enkianthus, sect. Andromedina, sect. Enkiantella and sect. Meisteria). Among them, sect. Enkianthus is monophyletic according to phylogenetic analyses (Tsutsumi and Hirayama 2012). However, due to the variable morphology in this genus (Hsu 1982), classification of some species, especially those with wide distribution range, remains controversial.

**Figure 1.**
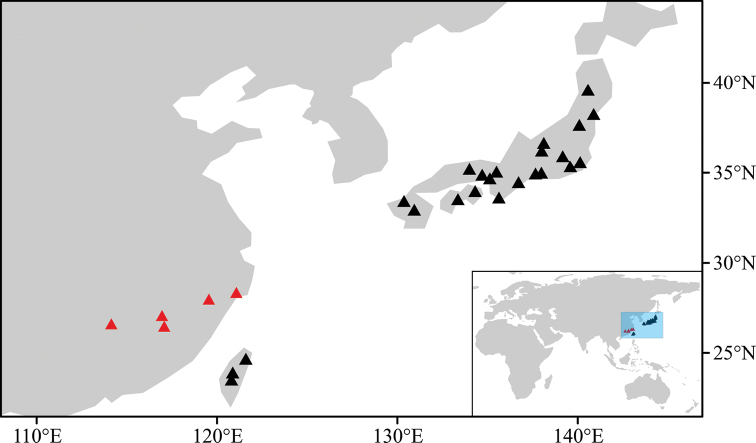
Distribution of *Enkianthusperulatus* based on specimen records and our field investigation. Black triangles indicate *E.perulatus*; red triangles indicate *E.calophyllus* (= *E.perulatus*).

**Figure 2.**
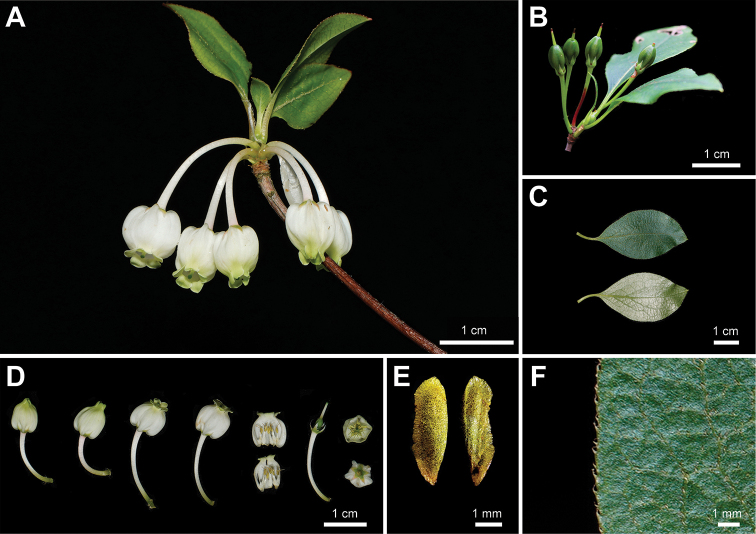
Morphology of *Enkianthuscalophyllus* (= *E.perulatus*) **A** flowering branch **B** fruiting branch **C** leaves **D** flowers **E** seeds **F** leaf margin. **A–F** photographed by H. Liang.

**Figure 3.**
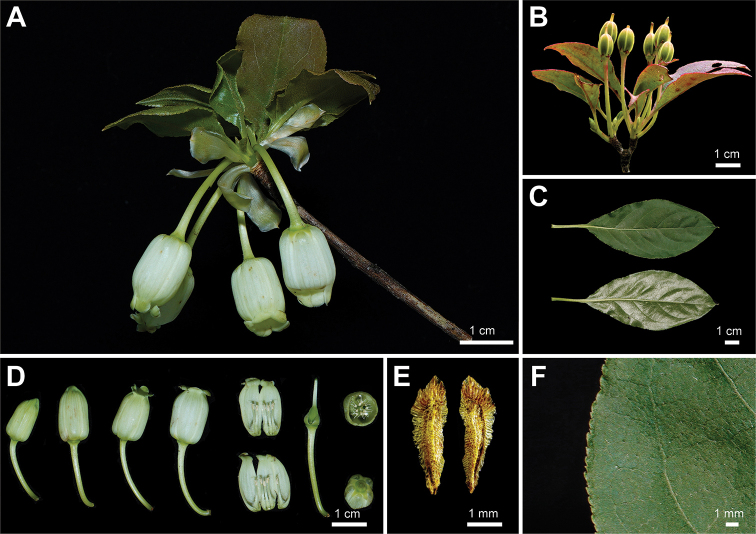
Morphology of *Enkianthusserrulatus***A** flowering branch **B** fruiting branch **C** leaves **D** flowers **E** seeds **F** leaf margin. **A–F** photographed by H. Liang.

**Figure 4.**
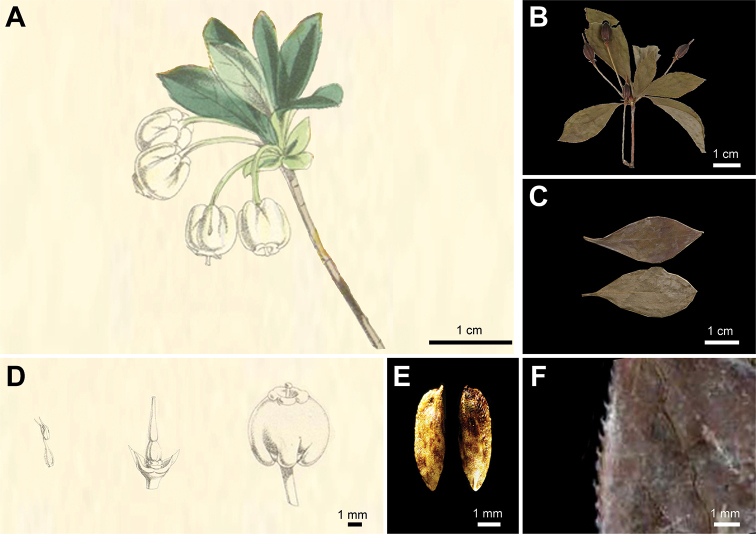
Morphology of *Enkianthusperulatus***A** flowering branch (Hooker 1870) **B** fruiting branch (USF 121400) **C** leaves (K-000780276) **D** flowers (Hooker 1870) **E** seeds **F** leaf margin (K-000780276). **E** photographed by H. Liang.

During the past years, we have found several unique *Enkianthus* populations in montane areas of Zhejiang and Jiangxi Province in China (Fig. 1). These plants are 1–3 m tall, white urceolate flowers with distinct basal gibbosities, rhombic-elliptic leaves and erect capsule. After scrutinizing the protologue and type specimens, we found that our collections matched the description of *E.calophyllus* T.Z. Hsu exactly (Fig. 2, Suppl. material 1: fig. S1B; Hsu 1985). When Fang and Stevens (2005) treated *E.calophyllus* as a synonym of *E.serrulatus* (E.H. Wils.) C.K. Schneid. (Fig. 3, Suppl. material 1: fig. S1C) in *Flora of China* (FOC), flowering specimens of *E.calophyllus* were lacking (Fang and Stevens 2005). However, our collections and *E.calophyllus* differ from *E.serrulatus* by urceolate corollas with distinct basal gibbosities, margin with ciliate, smaller fruits (0.5–0.7 cm × 0.3–0.4 cm) and seeds without distinct wings (Figs 2, 3; Schneider 1911; Hsu 1985), and such characters were described for *E.perulatus* C.K. Schneid. from Japan (Fig. 4, Suppl. material 1: fig. S1A; Schneider 1911). These observations raise a question about the taxonomic status of *E.calophyllus* and the identity of our collections. To identify our new collections and clarify the taxonomic status of *E.calophyllus*, morphological comparisons and molecular phylogenetic analyses were performed to study the taxonomic relationships amongst *E.calophyllus*, *E.perulatus*, *E.serrulatus* and our new collections.

## ﻿Materials and methods

### ﻿Morphological studies

Our collections and digital images of *Enkianthusperulatus*, *E.calophyllus* and *E.serrulatus* from the Chinese Virtual Herbarium (http://www.cvh.ac.cn/), the web of Plants of Taiwan (http://tai2.ntu.edu.tw), the Kingdonia (http://kun.kingdonia.org/) and the Global Biodiversity Information Facility (https://www.gbif.org/) were examined to test whether there are significant differences in leaves and fruits of the three species. The high-resolution images of those *Enkianthus* species were taken from specimens deposited at CDBI, CSFI, CSH, GA, GXMI, GZTM, JIU, JXAU, K, KUN, L, LGB, NAS, NTUF, P, PE, SYS, TAI, USF, and ZY. We randomly selected three well-preserved leaves and/or fruits from each specimen (28 specimens of *E.serrulatus*, 18 of *E.calophyllus* and 15 of *E.perulatus*). Among them, nine specimens of *E.calophyllus* and seven of *E.serrulatus* were collected by ourselves. Eight morphological characters, i.e., leaf length, leaf width, ratio of leaf length/width, pedicel length, fruit length, fruit width, ratio of fruit length/width and carpopodium length, were measured in this study. ImageJ (Rasband 1997) was applied to the examination of the focal characters.

ANOVA was performed to test the significance of pairwise difference of eight characters using SPSS 26. Principal Component Analysis (PCA) was performed in ORIGIN 2021 to investigate the morphological variations among *E.perulatus*, *E.calophyllus* and *E.serrulatus*. Morphological analysis was not carried out for flowers and seeds, because there were only a few specimens available for analysis.

### ﻿Sample collection, DNA extraction, PCR amplification, and sequencing

We collected 19 samples from 13 populations (1–3 individuals per population) of five *Enkianthus* species (Suppl. material 1: table S1). Of these, 17 samples belonged to species of sect. Enkianthus, i.e., six of *E.calophyllus*, four of *E.serrulatus*, four of *E.perulatus*, and three of *E.quinqueflorus* Lour. (Suppl. material 1: table S1). Two species of sect. Enkiantella, *E.chinensis* Franch. and *E.deflexus* (Griff.) Schneid. were also collected. Based on previous studies (Tsutsumi and Hirayama 2012), we downloaded nuclear ribosomal internal transcribed spacer (nrITS) and plastid DNA markers of other *Enkianthus* species from the National Center for Biotechnology Information’s (NCBI; http://www.ncbi.nlm.nih.gov/) nucleotide database (Suppl. material 1: table S1). In addition, we selected species of genera *Rhododendron*, *Vaccinium* and *Clethra* as outgroups to carry out the phylogenetic analysis of sect. Enkianthus according to previous studies (Kron et al. 2002; Liu et al. 2014). Voucher specimens are deposited in the Herbarium of Jiangxi Agricultural University (JXAU). Total genomic DNA were extracted from the silica-dried leaves using a modified cetyltrimethylammonium bromide method (Doyle and Doyle 1987). Six chloroplast DNA (cpDNA) regions (*psb*A-*trn*H, *rpL*32-*trn*L, *trn*L-*trn*F, *rps*16-*trn*Q, *psb*J-*pet*A and *mat*K) (Taberlet et al. 1991; Sang et al. 1997; Shaw et al. 2007) and nrITS (Sun et al. 1994) in 19 individuals from 13 populations of *Enkianthus*, were PCR amplified and sequenced. The PCR amplification protocols followed Cheng et al. (2021), and primers are listed in supplementary (Suppl. material 1: table S2). Newly generated sequences in this study are deposited in GenBank (Suppl. material 1: table S1).

### ﻿Phylogenetic analysis with cpDNA and nrDNA sequence data

The matrices of DNA sequences were aligned using MAFFT v.7 (Katoh and Standley 2013), and improved manually using BioEdit 7.0.9 (Hall 1999). Bayesian inference (BI) and maximum likelihood (ML) were used for phylogenetic analysis on the CIPRES Science Gateway 3.3 (www.phylo.org; Miller et al. 2015) with the best-fit model of DNA substitution estimated by jModelTest v.2.1.4 (Darriba et al. 2012). The alignments of nrITS, *mat*K and the concatenated plastid DNA (*psb*A-*trn*H + *rpL*32-*trn*L + *trn*L-*trn*F + *rps*16-*trn*Q + *psb*J-*pet*A + *mat*K) were analyzed with GTR + G, GTR and GTR + G + I model, separately. We reconstructed a *mat*K phylogeny of *Enkianthus* because the other five chloroplast DNA regions were sequenced only in a subset of species. Bayesian analysis was constructed using MrBayes v.3.2.7 (Ronquist et al. 2012). We performed two independent BI runs with one cold and three heated chains for 10,000,000 Markov chain Monte Carlo generations. We sampled trees every 1,000 generations and discarded the first 25% generations as burn-in. ML analysis was conducted by RAxML-HPC (Stamatakis 2014) with 1000 bootstrap replications.

## ﻿Results and discussion

### ﻿Morphological analyses

**Figure 5.**
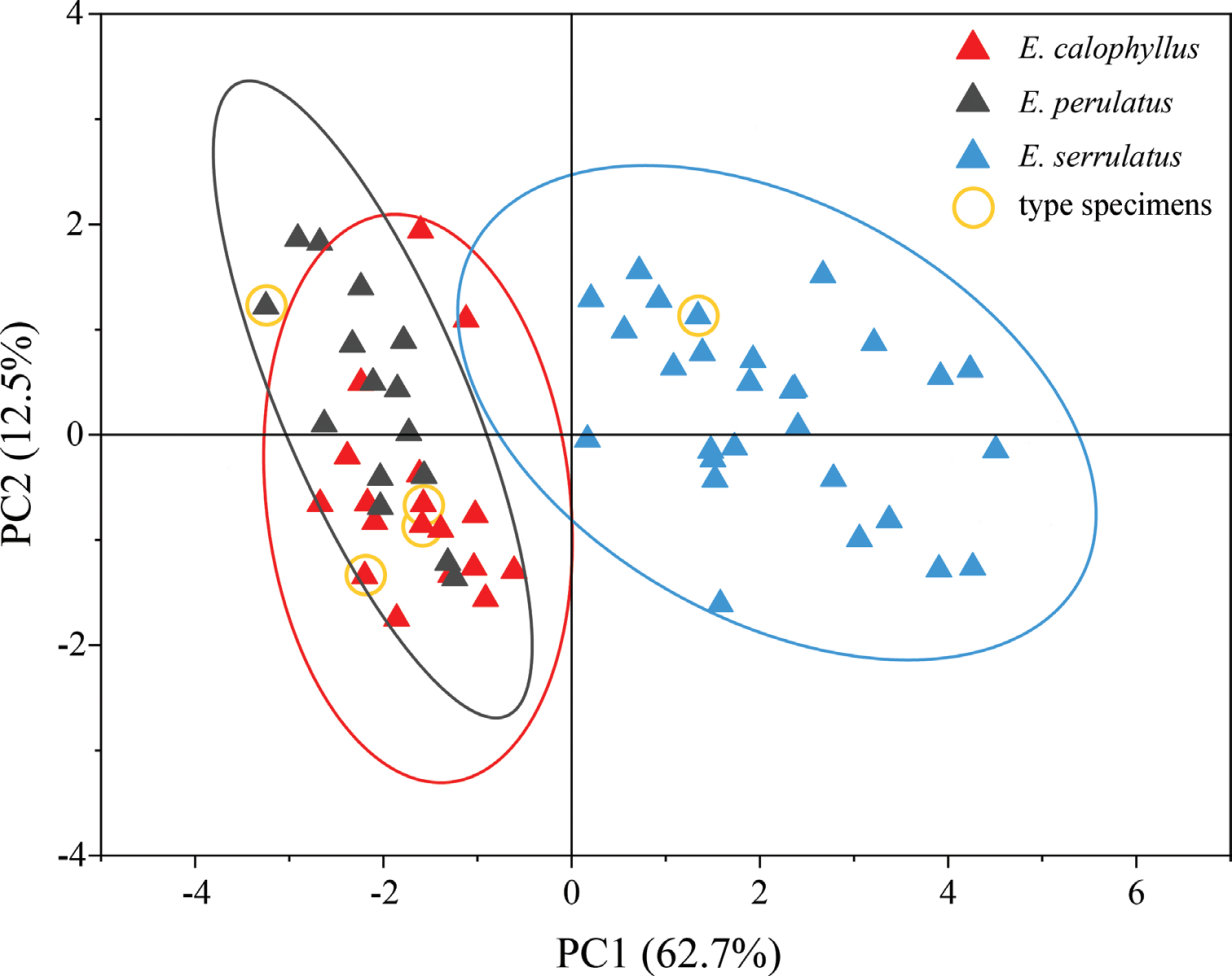
The Principal Component Analysis (PCA) plot for the morphological variations amongst *Enkianthusperulatus*, *E.calophyllus* (= *E.perulatus*) and *E.serrulatus*. Red, gray and blue triangles represent *E.perulatus*, *E.calophyllus* and *E.serrulatus*, respectively. Yellow circles indicate type specimens. The confidence ellipse level is 95%.

**Figure 6.**
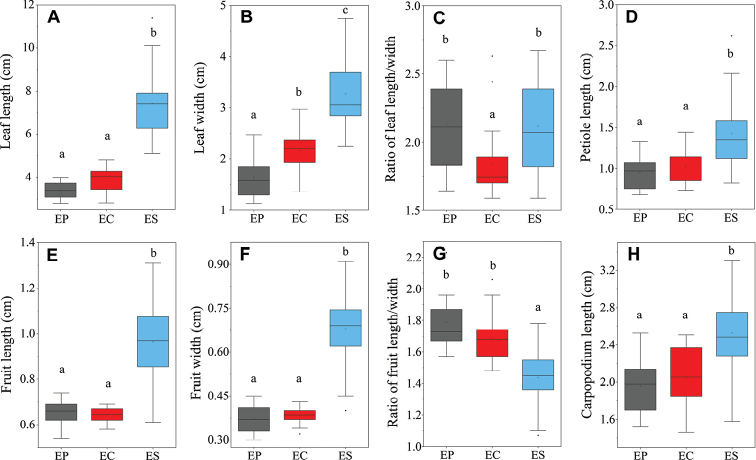
Box plots of the character comparisons amongst *Enkianthusperulatus* (EP), *E.calophyllus* (= *E.perulatus*; EC) and *E.serrulatus* (ES) **A** leaf length **B** leaf width **C** ratio of leaf length/width **D** pedicel length **E** fruit length **F** fruit width **G** ratio of fruit length/width **H** carpopodium length. The different lowercases on the top of the vertical line indicate significant differences (*P* < 0.05) between the three taxa.

Principal Component Analysis (PCA) showed that our collections of *Enkianthuscalophyllus* clustered with their type specimens and the 95% confidence ellipse of *E.calophyllus* intersected marginally that of *E.serrulatus*; however, the ellipse of *E.calophyllus* almost overlapped with that of *E.perulatus* (Fig. 5). In addition, pairwise comparisons of the eight morphological traits among *E.perulatus*, *E.calophyllus* and *E.serrulatus* (ANOVA analysis) showed there were significant differences between *E.calophyllus* and *E.serrulatus* (Fig. 6), but no significant difference between *E.calophyllus* and *E.perulatus* in all compared traits except for leaf width and ratio of leaf length/width (Fig. 6). Last but not least, morphological observation also found that *E.calophyllus* was almost the same as *E.perulatus*, but differs from *E.serrulatus* in flowers and seeds (Table 1). These results suggest that *E.calophyllus* should be conspecific with *E.perulatus* rather than *E.serrulatus*. The morphological description and comparison are elaborated in Table 1.

**Table 1.** Comparisons of morphological characters amongst *Enkianthusperulatus*, *E.calophyllus* and *E.serrulatus*.

Characters	*E.perulatus*	*E.calophyllus*	*E.serrulatus*
Habit	shrubs deciduous, 1–2 m tall	shrubs deciduous, 1–3 m tall	shrubs or small tree, deciduous, 3–6 m tall
Texture of leaf	Papery	Papery	papery or thickly papery
Petiole	0.5–1.2 cm	0.5–1.4 cm	0.7–2.1 cm
Leaf blade	oblong, obovate-oblong; 2–4 (–5) cm × 0.8–2.0 cm	rhombic-elliptic, elliptic; 2.5–5.0 cm × 1.5–3.0 cm	elliptic, oblong-elliptic or obovate-elliptic; 6–9 (–11) cm × 3–4 (–5) cm
Leaf margin	margin with ciliate	margin with ciliate	margin without ciliate
Inflorescence	umbellate, 1–5-flowered	umbellate, 1–5-flowered	umbellate, 2–6 (–9)-flowered
Corolla	urceolate with distinct basal gibbosities; white; 0.6–0.7 cm × 0.5–0.7 cm	urceolate with distinct basal gibbosities; white; 0.6–0.8 cm × 0.5–0.8 cm	oblong-urceolate without distinct basal gibbosities; greenish-white; 1.3–1.6 cm × 0.8–1.0 cm
Length/width of Corolla	1.0–1.3	1.0–1.4	1.5–2.0
Pedicel	1–2 cm	1.4–2.2 cm	2–3 cm
Fruit	capsule erect, oblong, 0.6–0.7 cm × 0.3–0.4 cm	capsule erect, oblong, 0.5–0.7 cm × 0.3–0.4 cm	capsule erect, oblong, 0.8–1.2 cm × 0.5–0.8 cm
Seed	without distinct wings	without distinct wings	with distinct wings
Distribution	Taiwan China, Japan (Honshu, Shikoku and Kyushu)	China (Zhejiang, Fujian, Jiangxi)	China (Jiangxi, Hubei, Hunan, Guangdong, Guangxi, Guizhou, Chongqing, Sichuan, Yunnan)
Altitude	200–1600 m	600–1200 m	800–1800 m

### ﻿Phylogenetic relationships

**Figure 7.**
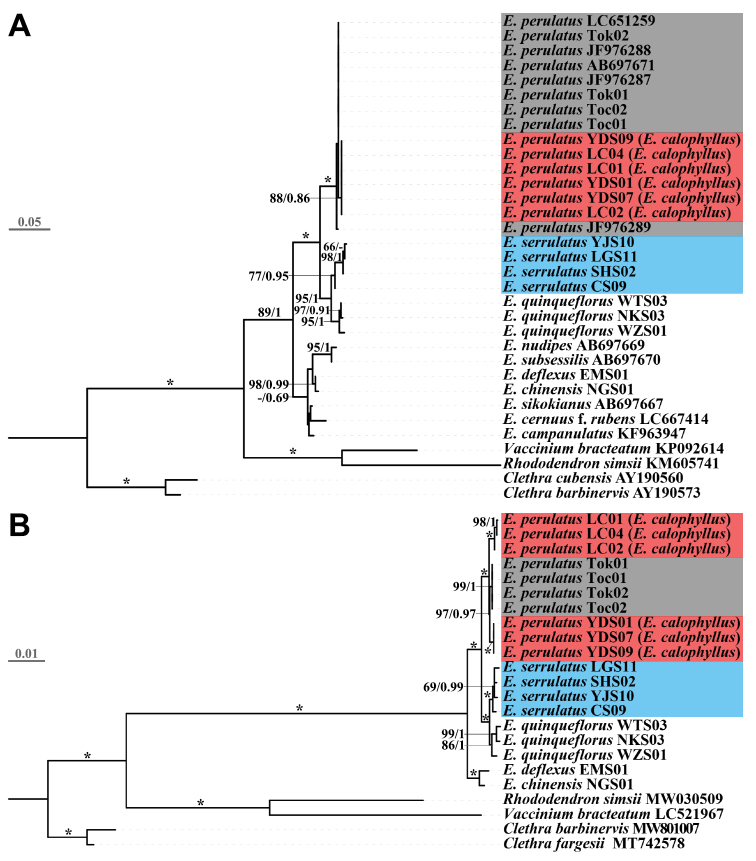
Phylogenetic relationships of *Enkianthus***A** phylogenetic tree based on nuclear DNA loci (ITS) **B** phylogenetic tree based on six plastid markers (*psb*A-*trn*H, *rpl*32-*trn*L, *trn*L-*trn*F, *rps*16-*trn*Q, *psb*J-*pet*A and *mat*K). Numbers and asterisksa above branches are Maximum Likelihood bootstrap values / Bayesian posterior probability (> 50%). Asterisks indicate that the support values are 100%. The phylogenetic positions of *E.perulatus*, *E.calophyllus* (= *E.perulatus*) and *E.serrulatus* are highlighted in red, grey and blue, respectively.

Alignment length of nrITS sequences based on 10 species (*E.calophyllus* = *E.perulatus*) of *Enkianthus* (approx. 83% species of *Enkianthus*, Fang and Stevens 2005) is 595 bp, including 75 variable sites and 60 parsimony informative sites. Alignments of *mat*K consisting of the same 10 species contain 755 constant sites, 24 variable sites and 9 parsimony informative sites. The concatenated length for six plastid DNA fragments based on five species of *Enkianthus* is 4,768 bp, and the matrix contains 124 variable sites and 85 parsimony informative sites in total. Phylogenetic analyses based on nrITS or *mat*K supported that sect. Enkianthus was a monophyletic clade. Although the nrITS tree showed that six accessions of *E.calophyllus* form a monophyletic clade (bootstrap value, BS = 88, Bayesian posterior probability, PP = 0.86; Fig. 7A), this clade was nested within *E.perulatus* (BS = 100, PP = 1; Fig. 7A). Notably, *E.calophyllus* intermingled with *E.perulatus*, forming a highly supported clade in the *mat*K tree (BS = 97, PP = 0.97; Suppl. material 1: fig. S2). Furthermore, the six plastid DNA tree supported that the monophyly of *E.perulatus* and *E.calophyllus* was recovered again (BS = 100, PP = 1; Fig. 7B), and *E.calophyllus* is paraphyletic with respect to *E.perulatus* (Fig. 7B). In all the trees, *E.serrulatus* clustered with *E.quinqueflorus* rather than with *E.perulatus*.

Taken together, we propose that *E.calophyllus* should be recognized as a new synonym of *E.perulatus* rather than the synonym of *E.serrulatus* as suggested by Fang and Stevens (2005). In addition, *E.serrulatus* together with *E.quinqueflorus* may represent a well differentiated lineage relative to *E.perulatus*.

### ﻿Taxonomic treatments

#### ﻿ Enkianthus perulatus

Taxon classificationPlantaeEricalesEricaceae

(Miq.) C.K. Schneid

B9EA57AB-40A9-50F2-B668-F008FAF1EC10

Andromeda perulata Miq., Ann. Mus. Bot. Lugduno-Batavi 1: 31. 1863. Basionym. Type: Japan. *W. Botanicus 57* (holotype: L-0007044!, Suppl. material 1: fig. S1A).
Enkianthus japonicus Hook. f., Bot. Mag. 96: 5822. 1870. Type: Japan. 1860, *R. Alcock s.n.* (holotype: K-000780276!). ≡ Enkianthusperulatusvar.japonicus (Hook. f.) Nakai, J. Jap. Bot. 12(12): 896. 1936. ≡ Enkianthusperulatusf.japonicus (Hook. f.) Kitam., Acta Phytotax. Geobot. 25: 36. 1972.
—Enkianthustaiwanianus S.S. Ying, Quart. J. Chin. Forest. 9: 145. 1976. Type: China. Taoyuan City, Peichiatienshan, 1976, *Ying 5301* (holotype: NTUF-F00006903!; isotype: NTUF-F00008372, NTUF-F00008372, NTUF-F00008372, NTUF-F00008372!). ≡ Enkianthusperulatusvar.taiwanianus (S.S. Ying) Y.C. Liu, Tr. Taiwan 514. 1988.
—Enkianthuscalophyllus T.Z. Hsu, Acta Bot. Yunnan. 7(2): 151–152. 1985. syn. nov. Type: China. Zhejiang: Jingning County, 16 May 1959, *S.Y. Zhang 5286* (holotype: PE-00005629!, Suppl. material 1: fig. S1B; isotype: NAS-00063024!, KUN-0001289!).

##### Description.

Deciduous shrubs up to 3 m tall. Branchlets terete, glabrous. Leaves alternate on young shoots, generally 4–6 crowded at apex of branchlets; petioles 0.5–1.4 cm long; blades papery, oblong, obovate-oblong, rhombic-elliptic or elliptic, 2–5 cm long, 0.8–3.0 cm wide, apex acuminate, mucronate, base attenuate-cuneate or cuneate, margin ciliate, upper surface glabrous except sparsely pubescent on midrib, lower surface villous along lower part of midrib. Inflorescences terminal, umbellate, 1–5-flowered, pendulous. Pedicels 1.0–2.2 cm long, glabrous, reflexed at flowering, straight at fruiting. Bracts lanceolate, white or green, 0.8–1.8 cm long, caducous. Calyx lobes triangular, 2–4 mm long, glabrous, deeply 5-lobed. Corollas urceolate, with distinct basal gibbosities, white, 6–8 mm long, 5–8 mm wide, shallowly 5-lobcd; lobes widely ovate, obtuse, much reflexed. Stamens 10, ca. 2/3 as long as corolla; filaments villous at base, anthers with 2 awns on upper dorsal side. Ovaries glabrous. Capsules erect, oblong, 5-ridged, 5–7 mm long, 3–4 mm wide. Seeds compressed, narrowly oblong, ca. 4 mm long, 1 mm wide, with marginal-like ridges, without distinct wings.

##### Phenology.

Flowering from April to May; fruiting from May to November.

##### Distribution and habitat.

*Enkianthusperulatus* is distributed in China (Zhejiang Fujian, Jiangxi and Taiwan) and Japan (Honshu, Shikoku and Kyushu) (Fig. 1). It grows on open rocky slopes, mountain slopes, cliffs, serpentine area, by roadsides or at forest margins at altitudes of 200–1600 m.

##### Additional specimens examined.

China. **Zhejiang**: Yueqing City, Yandangshan, 6 April 2015, *X.Y. Ye 2015040609* (CSH); *ibidem*, 20 May 2019, *H. Liang LSBZ*-*259* (JXAU). **Fujian**: Taining County, Xinqiao Town, 16 June 1978, *G.L. Cai* 445 (KUN). **Jiangxi**: Jinggangshan City, Jinggang Mountains, 15 July 1965, *S.K. Lai et al*. *4466* (LBG); Lichuan County, Huixianfeng, 20 October 1985, *S.K. Lai & D.F. Huang 473* (LBG); *ibidem*, 16 November 2021, *H. Liang 088* (JXAU). **Taiwan**: Taibei City, Tunlu, 14 April 1935, *T. Suzuki 19235* (TAI); Taibei City, Lupeishan, 16 April 1991, *Y.B. Cheng & T.S. Hsieh 1202* (TAI); Taoyuan City, Peichatienshan, 28 September 1984, *R.T. Li 3532* (TAI); Chiayi City, Alishan, 5 April 1982, *Y.F. Chen 4604* (TAI), *ibidem*, 17 May 1982, *Y.F. Chen 4902* (TAI). Japan. **Honshu**: Wakayama-Prefecture, Ohdaigahara Mountains, 23 May 1925, *S. Saito* (PE); Shizuoka-Prefecture, Tagata-gun, Sanagi Mountains, 24 April 1952, *M. Furuse 24733* (PE); Nagano-Prefecture, Iida-shi, 27 April 1962, *M. Furuse 39658* (PE); Aichi-Prefecture, Minami-shitara-gun, 22 April 1978, *M. Furuse 12532* (PE); Aichi-Prefecture, Shinshiro-shi, 6 August 1978, *M. Furuse 13039* (PE).

##### Additional specimens of *Enkianthusserrulatus* examined.

China. **Guangxi**: Debao County, 25 April 1977, *D. Fang et al. 3-219* (GXMI); Longsheng County, 24 August 2018, *H. Liang LSBZ-218* (JXAU); Longlin County, 17 May 1977, *T.H. Wei 3-0606* (GXMI); Xingan County, 26 July 1997, *G.Z. Li 15137* (PE). **Guizhou**: Chishui City, 24 May 2020, *H. Liang LSBZ-297* (JXAU); Leishan County, 13 June 2020, *H. Liang LSBZ-323* (JXAU); Songtao County, 22 July 1959, *T.P. Zhu et al. 1592* (KUN); Suiyang County, 11 May 2010, *Y.F. Zhou KKS101197* (ZY); Zhengan County, 14 October 2014, *H.W. Zhang 520324141014031LY* (GZTM). **Hubei**: Lichuan County, 7 October 1980, *B. Bartholomew et al. 2014* (PE); Tongshanx County, 14 May 2017, *H.Y. Zhan et al. LXP5905* (LBG); Yichang City, 19 March 2017, *D.G. Zhang et al. ZCJ170319117* (JIU). **Hunan**: Sangzhi County, 4 August 2017, *Z.Y. Zhang et al. LSBZ-142* (JXAU); Xinning City, 5 July 2017, *Z.K. Liu LSBZ-135* (JXAU); Yongshun County, 3 August 2017, *Z.Y. Zhang et al. LSBZ-138* (JXAU); Zhangjiajie City, 11 September 2015, *H. Zhou & D.S. Zhou 15091113* (CSFI). **Jiangxi**: Jinggangshan City, 27 August 2020, *Y.F. Liu LSBZ-365* (JXAU); Luxi County, 23 June 1984, *M.X. Nie 113* (LBG); Suichuan County, 24 June 2016, *Z.C. Liu et al. Lxp-13-18312* (SYS); Wuning County, 22 May 2014, *Y.H. Zhan et al. LXP0912* (LBG). **Sichuan**: Hechuan City, 1934, *D.J. Yu 3112* (PE); Leibo County, 3 July 1983, *Q.S. Zhao & Z.J. Zhao 121212* (PE); Xuyong County, 17 September 2013, *W.B. Ju & H.N. Deng HGX13668* (CDBI). Yunnan: Maguan County, 31 July 1961, *S.G. Wu 3597* (KUN); Suijiang County, 4 May 1973, *B.X. Sun 0112* (KUN). **Chongqing**: Fengjie County, 28 June 1958, *M.Y. Fang 24515* (NAS); Shizhu County, 2 June 1978, *W.H. Wang 1571* (CDBI).

## Supplementary Material

XML Treatment for Enkianthus perulatus

## Supplementary materials

Supplementary material 1 Supplementary data Data type figures and tables (word document) Explanation note Type specimen of *Enkianthusperulatus*, *E.calophyllus* and *E.serrulatus*. Phylogenetic relationships of *Enkianthus* based on *mat*K. Sample collection information and GenBank accessions in this study. Sequences of primers used for PCR amplification and sequencing. https://binary.pensoft.net/file/774090This dataset is made available under the Open Database License (http://opendatacommons.org/licenses/odbl/1.0/). The Open Database License (ODbL) is a license agreement intended to allow users to freely share, modify, and use this Dataset while maintaining this same freedom for others, provided that the original source and author(s) are credited.Hua Liang, Lu Jiang, Danqi Li, Yi Yang, Dengmei Fan, Zhiyong Zhang
