# Supplementary material for: ﻿A new synonym of Enkianthusperulatus (Ericaceae) in East Asia, based on morphological and molecular evidence
Source: PhytoKeys. 2022 Nov 25;214:61–74. doi: 10.3897/phytokeys.214.94294 (PMC9836438; doi:10.3897/phytokeys.214.94294)
Supplement: Supplementary material 1 — Supplementary data [file phytokeys-214-061_article-94294__-s001.docx]

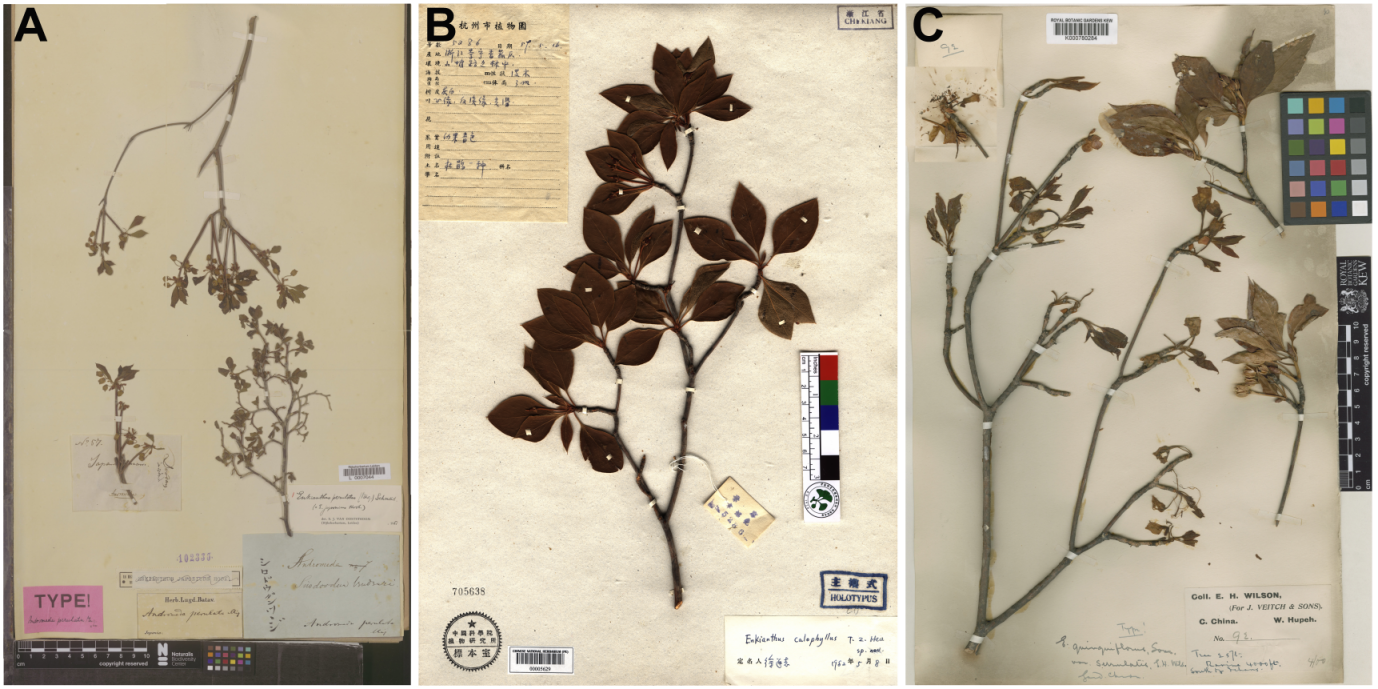


**Figure S1.** Type specimen of *Enkianthus perulatus*, *E. calophyllus* and *E. serrulatus*. A, holotype of *E. perulatus* (L-0007044); B, holotype of *E*. *calophyllus* (PE-00005629); C, holotype of *E*. *serrulatus* (K-000780284).


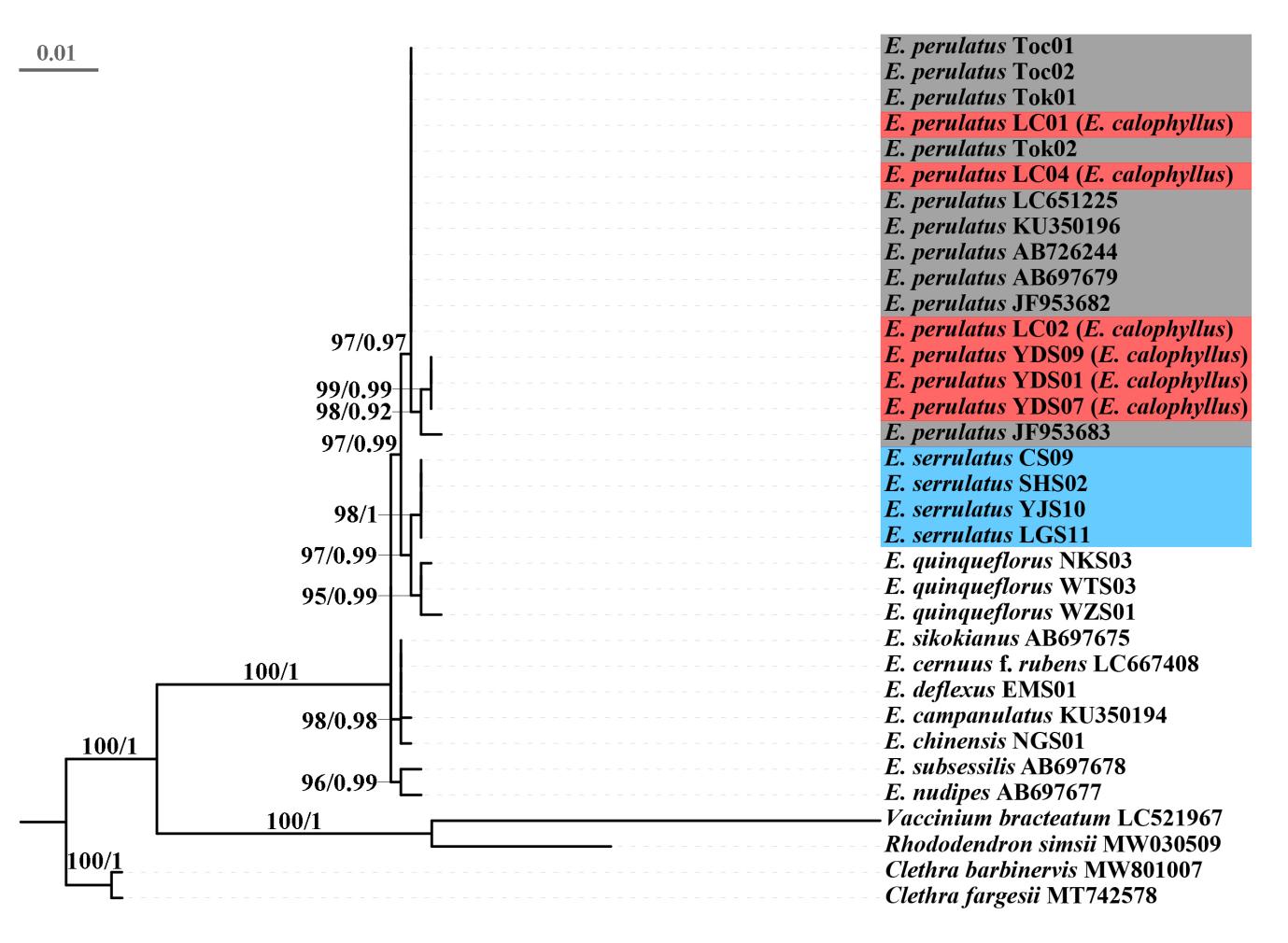


**Figure S2.** Phylogenetic relationships of *Enkianthus* based on *mat*K. Numbers above branches are Maximum Likelihood bootstrap values /Bayesian posterior probability (> 50%). The phylogenetic position of *E. perulatus*, *E. calophyllus* (= *E*. *perulatus*) and *E. serrulatus* are highlighted in red, grey and blue, respectively.

**Table S1.** Sample collection information and GenBank accessions in this study.

| **Species** | **Source** | **Samples ID** | **GenBank number** | | | | | | |
| --- | --- | --- | --- | --- | --- | --- | --- | --- | --- |
|  |  |  | ***psb*A-*trn*H** | ***rpl*32-trnL** | ***trn*L-F** | ***rps*16-*trn*Q** | ***psb*J-*pet*A** | ***mat*K** | **ITS** |
| *E. perulatus* | Tokyo, Japan | Tok01 | OP024388 | OP024412 | OP024436 | OP024460 | OP024484 | OP024508 | OP002295 |
| *E. perulatus* | Tokyo, Japan | Tok02 | OP024389 | OP024413 | OP024437 | OP024461 | OP024485 | OP024509 | OP002296 |
| *E. perulatus* | Tochigi, Japan | Toc01 | OP024390 | OP024414 | OP024438 | OP024462 | OP024486 | OP024510 | OP002297 |
| *E. perulatus* | Tochigi, Japan | Toc02 | OP024391 | OP024415 | OP024439 | OP024463 | OP024487 | OP024511 | OP002298 |
| *E. perulatus* | GenBank |  | – | – | – | – | – | AB697679 | AB697671 |
| *E. perulatus* | GenBank |  | – | – | – | – | – | JF953682 | JF976288 |
| *E. perulatus* | GenBank |  | – | – | – | – | – | JF953683 | JF976289 |
| *E. perulatus* | GenBank |  | – | – | – | – | – | LC651225 | JF976287 |
| *E. perulatus* | GenBank |  | – | – | – | – | – | KU350196 | LC651259 |
| *E. perulatus* | GenBank |  | – | – | – | – | – | AB726244 | – |
| *E. calophyllus* | Yueqing, Zhejiang, China | YDS01 | OP024377 | OP024401 | OP024425 | OP024449 | OP024473 | OP024497 | OP002289 |
| *E. calophyllus* | Yueqing, Zhejiang, China | YDS04 | OP024378 | OP024402 | OP024426 | OP024450 | OP024474 | OP024498 | OP002290 |
| *E. calophyllus* | Yueqing, Zhejiang, China | YDS09 | OP024379 | OP024403 | OP024427 | OP024451 | OP024475 | OP024499 | OP002291 |
| *E. calophyllus* | Lichuan, Jiangxi, China | LC01 | OP024380 | OP024404 | OP024428 | OP024452 | OP024476 | OP024500 | OP002292 |
| *E. calophyllus* | Lichuan, Jiangxi, China | LC02 | OP024381 | OP024405 | OP024429 | OP024453 | OP024477 | OP024501 | OP002293 |
| *E. calophyllus* | Lichuan, Jiangxi, China | LC04 | OP024382 | OP024406 | OP024430 | OP024454 | OP024478 | OP024502 | OP002294 |
| *E. serrulatus* | Yongxiu, Jiangxi, China | YJS10 | OP024395 | OP024419 | OP024443 | OP024467 | OP024491 | OP024515 | OP002285 |
| *E. serrulatus* | Xinning, Hunan, China | SHS02 | OP024396 | OP024420 | OP024444 | OP024468 | OP024492 | OP024516 | OP002284 |
| *E. serrulatus* | Leishan, Guizhou, China | LGS11 | OP024397 | OP024421 | OP024445 | OP024469 | OP024493 | OP024517 | OP002283 |
| *E. serrulatus* | Chishui, Guizhou, China | CS09 | OP024398 | OP024422 | OP024446 | OP024470 | OP024494 | OP024518 | OP002282 |
| *E. quinqueflorus* | Longmen, Guangdong, China | NKS03 | OP024392 | OP024416 | OP024440 | OP024464 | OP024488 | OP024512 | OP002286 |
| *E. quinqueflorus* | Shenzhen, Guangdong, China | WTS03 | OP024393 | OP024417 | OP024441 | OP024465 | OP024489 | OP024513 | OP002287 |
| *E. quinqueflorus* | Wuzhishan, Hainan, China | WZS01 | OP024394 | OP024418 | OP024442 | OP024466 | OP024490 | OP024514 | OP002288 |

| **Species** | **Source** | **samples name** | **GenBank number** | | | | | | |
| --- | --- | --- | --- | --- | --- | --- | --- | --- | --- |
|  |  |  | ***psb*A-*trn*H** | ***rpl*32-trnL** | ***trn*L-F** | ***rps*16-*trn*Q** | ***psb*J-*pet*A** | ***mat*K** | **ITS** |
| *E. chinensis* | Langao, Shaanxi, China | NGS01 | OP024385 | OP024409 | OP024433 | OP024457 | OP024481 | OP024505 | OP002301 |
| *E. deflexus* | Emeishan, Sichuan, China | EMS01 | OP024386 | OP024410 | OP024434 | OP024458 | OP024482 | OP024506 | OP002302 |
| *E. campanulatus* | GenBank |  | – | – | – | – | – | KU350194 | KF963947 |
| *E. sikokianus* | GenBank |  | – | – | – | – | – | AB697675 | AB697667 |
| *E. cernuus* f. *rubens* | GenBank |  | – | – | – | – | – | LC667408 | LC667414 |
| *E. nudipes* | GenBank |  | – | – | – | – | – | AB697677 | AB697669 |
| *E. subsessilis* | GenBank |  | – | – | – | – | – | AB697678 | AB697670 |
| *Rhododendron simsii* | GenBank |  | MW030509 | MW030509 | MW030509 | MW030509 | MW030509 | MW030509 | KM605741 |
| *Vaccinium bracteatum* | GenBank |  | LC521967 | LC521967 | LC521967 | LC521967 | LC521967 | LC521967 | KP092614 |
| *Clethra barbinervis* | GenBank |  | MW801007 | MW801007 | MW801007 | MW801007 | MW801007 | MW801007 | AY190573 |
| *Clethra cubensis* | GenBank |  | – | – | – | – | – | – | AY190560 |
| *Clethra fargesii* | GenBank |  | MT742578 | MT742578 | MT742578 | MT742578 | MT742578 | MT742578 | – |

“–”, the sequence is unavailable.

**Table S2.** Sequences of primers used for PCR amplification and sequencing.

| Fragment | Forward primer (5’–3’) | Reverse primer (5’–3’) | Reference |
| --- | --- | --- | --- |
| *psb*A*-trn*H | GTTATGCATGAACGTAATGCTC | CGCGCATGGTGGATTCACAAATC | Sang et al. 1997 |
| *rpl*32*-trn*L | CAGTTCCAAAAAAACGTACTTC | CTGCTTCCTAAGAGCAGCGT | Shaw et al. 2007 |
| *trn*L*-trn*F | CGAAATCGGTAGACGCTACG | ATTTGAACTGGTGACACGAG | Taberlet et al. 1991 |
| *rps*16*-trn*Q | GTTGCTTTYTACCACATCGTTT | GCGTGGCCAAGYGGTAAGGC | Shaw et al. 2007 |
| *psb*J-*pet*A | ATAGGTACTGTARCYGGTATT | AACARTTYGARAAGGTTCAATT | Shaw et al. 2007 |
| *mat*K | TGGTTCAAACTATTCGCTACTG | ATAACAAACTTGCTCTTCCTCC | – |
| ITS | ACGAATTCATGGTCCGGTGAAGTGTTCG | TAGAATTCCCCGGTTCGCTCGCCGTTAC | Sun et al. 1994 |
